# Biomechanical Factors and Prevention Strategies for Sports-Related Muscle Injuries: A Narrative Review

**DOI:** 10.3390/bioengineering13040473

**Published:** 2026-04-17

**Authors:** Catalin Ionite, Lucian Indrei, Andrei Gheorghiță, Bogdan Caba, Marius Turnea, Irina Duduca, Cezar Mucileanu, Iustina Condurache, Mariana Rotariu

**Affiliations:** 1 Grigore T. Popa University of Medicine and Pharmacy Iasi, 700454 Iasi, Romania; 2 Faculty of Machines Manufacturing and Industrial Management, “Gheorghe Asachi” Technical University of Iasi, 700050 Iasi, Romania

**Keywords:** muscle injury prevention, sports biomechanics, neuromuscular training

## Abstract

Sports-related muscle injuries represent a major challenge in both recreational and professional sports, accounting for a substantial proportion of time-loss injuries and frequently leading to recurrent episodes. The aim of this narrative review was to analyze the biomechanical and neuromuscular mechanisms involved in the occurrence of muscle injuries and to synthesize evidence-based prevention strategies reported in the scientific literature. The literature search was conducted in the Web of Science database using the keyword “muscle injury prevention”, focusing on studies published between 2010 and 2025. The analyzed literature indicates that muscle injuries are strongly associated with eccentric contractions at long muscle lengths, neuromuscular fatigue, strength imbalances, impaired lumbopelvic stability, and inadequate load management. Preventive strategies based on biomechanical principles, particularly eccentric strength training, neuromuscular training programs, and core stability exercises, have demonstrated consistent effectiveness in reducing injury incidence and recurrence rates across multiple sports disciplines. In addition, emerging technological approaches, including wearable sensors and machine learning models, show promising potential for injury risk prediction and individualized prevention strategies.

## 1. Introduction

Sports-related injuries represent a major concern for athletes, medical staff, and performance specialists, with muscle injuries accounting for a substantial proportion of time-loss episodes in both recreational and elite sports. Epidemiological studies published over the past decades indicate that muscle injuries comprise approximately 10–17% of all sports-related injuries, with considerably higher rates reported in disciplines characterized by sprinting, rapid accelerations, and high mechanical demands, such as football, rugby, athletics, and basketball [1,2,3,4,5,6,7]. Of particular concern is the high recurrence rate, frequently reported between 30% and 40%, underscoring the persistent and multifactorial nature of these injuries [5,7,8,9].

The increasing incidence of muscle injuries observed between 2010 and 2025 has been attributed to the interaction of multiple factors, including intensified training loads, high competition density, early sport specialization, and suboptimal recovery strategies [2,4,9]. Cohort studies conducted in team sports, particularly football and athletics, have demonstrated that injury incidence is significantly higher during competition compared with training sessions, highlighting the role of acute mechanical stress and neuromuscular fatigue in injury occurrence [10,11]. These findings support the need for a transition from reactive management approaches toward prevention strategies grounded in the underlying mechanisms of injury development.

Traditional preventive approaches, such as isolated stretching or generalized strength training, have demonstrated limited effectiveness when applied as standalone interventions [3,12,13]. In contrast, recent research increasingly supports prevention models grounded in biomechanical and neuromuscular principles, targeting muscle–tendon unit behavior, eccentric force production capacity, intermuscular coordination, and load management [14,15,16,17]. Numerous experimental and longitudinal studies have shown that deficits in eccentric strength, alterations in muscle architecture, impaired lumbopelvic control, and neuromuscular fatigue significantly increase susceptibility to muscle injuries, particularly at the level of the hamstrings and adductors [16,18,19,20].

In response to these findings, research in injury prevention has evolved toward integrated, biomechanically informed strategies that include eccentric strength exercises (such as the Nordic Hamstring Exercise), neuromuscular training, core stability exercises, and structured monitoring of training load [4,21,22,23,24]. The implementation of these programs has been consistently associated with reductions in muscle injury incidence ranging from 40% to 70%, as well as with significant decreases in recurrence rates [8,10,25,26]. The reproducibility of these outcomes across multiple sports disciplines and performance levels highlights the translational relevance of biomechanical research in the prevention of muscle injuries.

However, the existing literature remains fragmented, and data regarding biomechanical mechanisms, injury epidemiology, and the effectiveness of preventive interventions are often reported separately. Methodological variability, the lack of standardized injury definitions, and the heterogeneity of assessment protocols limit the direct clinical applicability of the findings [12,25,26]. In this context, the adoption of standardized reporting frameworks, such as the International Olympic Committee consensus on injury definitions and data collection procedures or other consensus statements used in sports injury epidemiology, may represent an important step toward reducing methodological heterogeneity and improving the consistency and clinical applicability of findings across studies [27,28]. From an orthopedic and trauma perspective, there is therefore a clear need for an integrated synthesis that correlates mechanical loading, neuromuscular control, and tissue adaptations with evidence-based prevention strategies.

In this context, the aim of the present narrative review is to analyze the biomechanical factors involved in the occurrence of sport-related muscle injuries and to synthesize effective prevention strategies based on scientific literature published between 2010 and 2025. By integrating epidemiological data, biomechanical mechanisms, and the outcomes of preventive interventions, this article seeks to provide a comprehensive framework for the development of prevention programs tailored to the specific demands of each sport.

## 2. Literature Search and Review Methodology

The literature search was conducted exclusively using the Web of Science database (Clarivate Analytics), employing “muscle injury prevention” as the primary search term. The use of this single keyword was a deliberate methodological choice aimed at increasing specificity and maintaining conceptual coherence within the analyzed literature. The search strategy was designed to identify relevant articles that explicitly address muscle injury prevention from biomechanical, orthopedic, and neuromuscular perspectives, while avoiding the inclusion of studies in which this topic was only marginally or indirectly discussed.

The initial search yielded a total of 7071 documents, including 5646 original research articles and 1203 review articles. To ensure consistency with the objective of the present review and to capture the temporal evolution of research and methodological trends in the field, a temporal filter was applied, and only studies published between 2010 and 2025 were retained for further analysis. This selection resulted in a refined dataset of 4955 documents, which were analyzed separately across three distinct time periods.

During the 2010–2014 period, 1061 documents were identified (856 original research articles and 160 review articles), of which 33 were included in the final analysis. For the 2015–2019 interval, 1602 documents were retrieved (1282 original articles and 248 reviews), with 25 studies meeting the eligibility criteria. Between 2020 and 2025, 2292 documents were identified (1786 original research articles and 458 review articles), of which 28 were included in the final synthesis.

**Figure 1:**
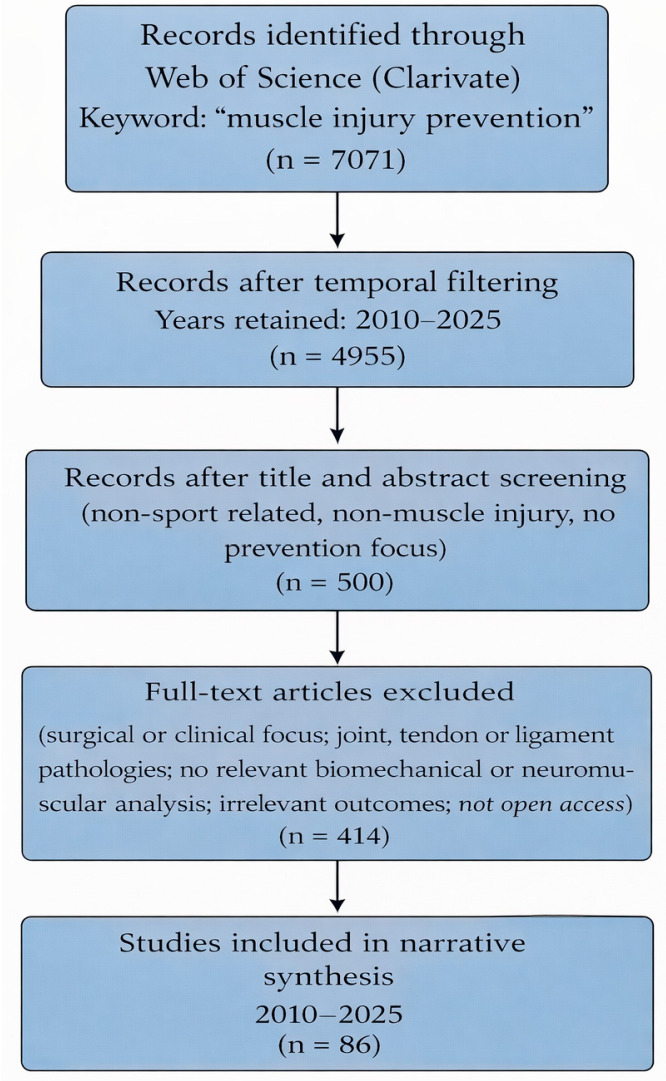
PRISMA-like flow diagram illustrating the literature search and selection process for the narrative review on muscle injury prevention using the Web of Science database (2010–2025).

The selection process (Figure 1) involved a stepwise screening of titles, abstracts, and subsequently full-text articles, with the aim of identifying studies that explicitly address muscle injury incidence, the biomechanical and neuromuscular factors involved in injury occurrence, and scientifically validated prevention strategies. The high number of articles excluded at the full-text stage can be explained by the fact that a substantial proportion of initially identified studies did not align with the specific objective of the present narrative review. These included studies primarily focused on surgical interventions, postoperative management, joint, tendon, or ligament pathologies, as well as studies centered on general rehabilitation, sports performance, or other musculoskeletal conditions without a direct link to muscle injury prevention. Additionally, studies lacking relevant biomechanical or neuromuscular analysis or not presenting scientifically validated preventive interventions were excluded. This approach enabled the development of a coherent narrative review grounded in relevant evidence published between 2010 and 2025, appropriate for analyzing the topic within the context of orthopedic and trauma biomechanics.

## 3. Epidemiology of Sports-Related Muscle Injuries

Sport-related muscle injuries represent a major musculoskeletal health concern, accounting for a substantial proportion of reported injuries in both elite and recreational sports. An analysis of the literature published between 2010 and 2025 indicates that muscle injuries comprise approximately 10–17% of all sports-related injuries, with considerable variation depending on the specific sport discipline, competitive level, and reporting methodology employed [2,3,4,5,6,29]. From both a clinical and biomechanical perspective, a particularly relevant aspect is the high recurrence rate, frequently reported between 30% and 40%, suggesting the persistence of biomechanical and neuromuscular risk factors following return to play [7,8,22].

### 3.1. Differences Across Sports Disciplines

The distribution of muscle injuries differs significantly across sports disciplines, depending on mechanical loading patterns and sport-specific functional demands.

In football, muscle injuries represent one of the most frequent causes of time loss, with an estimated prevalence ranging between 11% and 17% of all reported injuries, hamstring injuries being predominant [22,27,30]. Longitudinal studies report an average incidence of approximately 0.6 muscle injuries per player per season, with a substantial impact on time lost from competition [5,28,31].

In rugby and Australian football, sports characterized by frequent contact and explosive efforts, muscle injury incidence is reported to be considerably higher during competition compared to training. Reported values range from 0.27/1000 h in training to 5.6/1000 h in matches, reflecting increased exposure to acute mechanical stress [1,32].

In athletics, muscle and musculotendinous injuries account for up to 40–60% of all injuries, with distribution depending on the specific event. Sprint and hurdle events are predominantly associated with acute thigh injuries, whereas endurance events present a higher risk of chronic overload-related conditions [11,33,34].

In basketball, the incidence of muscle injuries has been reported to exceed that of ankle sprains, with values of approximately 2.3/1000 h, increasing significantly during competition [20,35].

In baseball, injury patterns are strongly influenced by repetitive high-velocity rotational movements and explosive sprint efforts. Epidemiological data from professional leagues indicate that abdominal and trunk muscle injuries are among the leading causes of time-loss, particularly in pitchers, with a documented increase in incidence over recent seasons [16]. Additionally, hamstring strains occur primarily during sprint initiation and base running, reflecting high eccentric loading at long muscle lengths [18,36]. Preventive interventions in elite players, particularly the Nordic Hamstring Exercise, have demonstrated substantial reductions in hamstring injury incidence, including complete absence of injury in compliant athletes compared with controls. These findings highlight the sport-specific relevance of posterior chain strengthening and trunk stability in baseball injury prevention programs [37,38].

In volleyball, although the overall incidence is lower, studies indicate that structured preventive programs may completely eliminate the occurrence of muscle injuries in certain cohorts [10,21].

In contrast, in recreational winter sports such as alpine skiing and snowboarding, injury patterns are influenced by high velocities, impact forces, technical errors, fatigue, and insufficient physical conditioning [39]. Epidemiological data indicate that these activities contribute substantially to the incidence of sports-related injuries, with reported rates of approximately 2.4–2.6 per 1000 activity days for skiing and 3.6–7.0 per 1000 activity days for snowboarding [40]. Major risk factors include lack of experience, low technical skill level, poor physical fitness, risk-taking behavior, adverse environmental and slope conditions, and the use of inappropriate or rented equipment, particularly among recreational participants. However, most prevention strategies remain focused on equipment, education, and behavioral measures, while exercise-based interventions are insufficiently investigated and not standardized in the current literature. Although physical conditioning, neuromuscular training, and core stability may contribute to injury reduction, no specific exercise protocols have been clearly defined for these sports. Therefore, training programs targeting lower limb musculature and trunk stability, including ski-specific simulation devices, may represent a relevant approach to improving neuromuscular control, balance, and adaptation to sport-specific loads.

In youth ice hockey, a sport characterized by high-speed skating, frequent collisions, and substantial mechanical demands, overall injury incidence has been reported to range from 11.7 to 34.4 per 1000 player-hours. Moreover, injuries occur markedly more often during matches than during training sessions, with match injury rates reported to be more than eight times higher, and in some cases up to 25 times higher, than those observed in training. This pattern reflects the greater exposure to acute physical contact and competitive stress during game situations [41].

In artistic gymnastics, a sport characterized by repetitive high-impact landings, complex acrobatic maneuvers, and substantial mechanical loading of both the upper and lower extremities, injury incidence has been reported to range from 1.5 to 9.2 injuries per 1000 training hours, depending on age group and competitive level. Moreover, overuse injuries account for nearly 60–70% of all reported cases, most commonly affecting the wrists, ankles, and lower back. This pattern reflects the cumulative effect of repetitive loading, technical demands, insufficient recovery, and the increased vulnerability of the musculoskeletal system during periods of growth and at higher levels of competition [42].

### 3.2. Anatomical Distribution of Sports-Related Muscle Injuries

From an anatomical perspective, the literature consistently indicates a clear predominance of muscle injuries involving the lower extremities, particularly the hamstrings, adductors, quadriceps, and gastrocnemius muscles.

Hamstring injuries are reported as the most frequent, accounting for approximately 12% to 34% of all muscle injuries, especially in sports that involve repeated sprinting and rapid acceleration [30,43]. Adductor and quadriceps injuries are commonly observed in sports characterized by abrupt changes of direction, whereas gastrocnemius injuries occur predominantly in activities requiring repetitive push-off actions [36,44].

### 3.3. Differences in Muscle Injury Incidence Between Competition and Training

A consistent finding reported in the analyzed literature is the significantly higher incidence of muscle injuries during competition compared with training sessions. In basketball, match incidence reaches values of 20.5/1000 h, compared to 1.3/1000 h during training [3]. Similar trends have been reported in football and rugby, where exposure to maximal intensity efforts and neuromuscular fatigue play a determining role [45].

### 3.4. Sex-Related and Competition-Level Differences

Sex and competitive level differences significantly influence the epidemiology of muscle injuries. In athletics, incidence has been reported to be higher in male athletes (~52/1000 athletes) compared with female athletes (~30/1000 athletes), a difference commonly attributed to biomechanical demands and training volume [12]. Nevertheless, sex-related variations in injury risk may also reflect intrinsic physiological and neuromuscular differences. Hormonal variations, particularly fluctuations in estrogen levels, have been associated with changes in ligament laxity and muscle–tendon mechanical properties, potentially affecting injury susceptibility. In addition, differences in neuromuscular control strategies, muscle activation patterns, and intersegmental coordination may contribute to variations in load distribution during high-intensity movements.

Moreover, higher competitive levels are associated with increased injury frequency and elevated recurrence rates, particularly in professional sports, where competition density and performance pressure are substantially greater [10].

## 4. Biomechanical and Neuromuscular Factors Contributing to Muscle Injury

Understanding the biomechanical and neuromuscular mechanisms underlying muscle injury occurrence is essential for the development of effective prevention strategies. The analyzed literature highlights that muscle injuries are not isolated events, but rather the result of a complex interaction between the mechanical properties of the muscle–tendon unit, strength imbalances, impaired neuromuscular control, and inadequate management of functional loading.

### 4.1. Muscle–Tendon Unit Mechanics

The mechanical properties of the muscle–tendon unit play a central role in the capacity of muscle tissue to tolerate the demands imposed by sporting activity. Numerous studies have demonstrated that muscle injuries frequently occur during eccentric contractions performed at long muscle lengths, a situation characteristic of the terminal phases of sprinting, decelerations, and rapid changes of direction [2,3,8,16]. Under these conditions, the mechanical tension exerted on muscle fibers and connective tissue structures exceeds the adaptive capacity of the tissue, predisposing to muscle strain and rupture [46].

In a recent narrative review, Andrews et al. analyzed the biomechanical mechanisms underlying hamstring injuries associated with high-speed running and reported that more than 80% of these injuries occur during the late swing phase of sprinting, when the posterior thigh musculature generates high eccentric forces to decelerate the lower limb [47]. The authors suggest that reduced tolerance to active fascicle lengthening, together with repeated mechanical loading, represents major biomechanical factors contributing to the occurrence of these injuries [48].

Muscle–tendon unit stiffness represents another key biomechanical determinant. Excessive stiffness limits the capacity to absorb mechanical energy, whereas insufficient stiffness reduces the efficiency of force transmission and increases strain on muscle fibers [19]. The included studies indicate that adaptations induced by eccentric training may optimize the functional stiffness of the muscle–tendon unit, thereby contributing to a reduced risk of injury [23].

In addition, muscle fascicle length has been identified as an important predictor of injury risk [49]. Shorter fascicles are associated with a reduced ability to tolerate elongation during eccentric phases, particularly at the level of the hamstrings [16,20]. Increases in fascicle length achieved through specific eccentric exercises, such as the Nordic Hamstring Exercise, have been correlated with decreased muscle injury incidence [8].

Pennation angle influences the relationship between force generated at the microscopic level and force transmitted to the tendon. Training-induced modifications of this parameter may affect the distribution of mechanical stress within the muscle and, consequently, susceptibility to injury [18].

### 4.2. Strength Imbalances and Force Ratios

Strength imbalances represent a major risk factor for the occurrence of muscle injuries, particularly in sports characterized by explosive movements. The analyzed literature highlights the role of the hamstrings-to-quadriceps strength ratio (H:Q), especially the eccentric component of hamstring strength, in maintaining functional knee stability [23]. A reduced H:Q ratio has been associated with an increased risk of both muscle and ligament injuries.

Bilateral strength asymmetries between the lower limbs have also been correlated with a higher incidence of injuries, particularly in team sports [2,7,42]. Significant differences in strength and neuromuscular control between limbs reflect asymmetric adaptations to loading and may lead to uneven distribution of mechanical stress [50].

In addition, quadriceps dominance relative to the posterior thigh musculature is frequently reported as a predisposing factor for muscle and joint injuries, particularly under conditions of neuromuscular fatigue [50,51]. These findings support the inclusion of eccentric training exercises and sport-specific functional assessments in injury prevention programs.

Recent research has begun to employ advanced data analysis methods to identify biomechanical biomarkers associated with injury risk [52]. In a study conducted on professional soccer players, the authors applied machine learning algorithms to analyze biomechanical variables related to muscle injuries and identified maximal hamstring strength and muscle stiffness as important predictors of injury occurrence [53]. These findings suggest that the functional assessment of muscle strength and the mechanical properties of muscle tissue may contribute to the identification of athletes at increased risk of injury.

### 4.3. Neuromuscular Control and Motor Coordination

Neuromuscular control and motor coordination represent key elements in maintaining musculoskeletal integrity. The analyzed studies indicate that deficits in muscle timing and anticipatory activation may compromise joint stability and increase strain on muscular tissues [54]. Delayed activation of stabilizing musculature reduces the capacity to dissipate external forces, thereby predisposing to injury [55].

Impaired frontal plane control, manifested as dynamic valgus, is associated with increased mechanical loading of the thigh musculature and joint structures, particularly in sports involving rapid changes of direction [11,47]. This biomechanical pattern is frequently correlated with insufficient neuromuscular control and strength imbalances.

In addition to classical biomechanical factors, several studies have also highlighted the role of cognitive factors in movement control. In an experimental study conducted on junior soccer players, the authors analyzed the effect of mental stress on movement kinematics during dynamic running tasks. The results showed that cognitive load can alter movement patterns and increase muscular tension, suggesting that the interaction between mental and biomechanical demands may influence the risk of injury [55].

Lumbopelvic stability plays a central role in the efficient transmission of force between body segments. Deficits at this level may alter kinetic chain mechanics and increase the risk of muscle injuries in the lower extremities [17,19]. Training programs incorporating core stability exercises have demonstrated positive effects in reducing muscle injury incidence [22,23,48].

### 4.4. Load Management and Fatigue in Muscle Injury Risk

Functional load management represents a major determinant of injury risk. The literature indicates that both overload and underload may increase susceptibility to muscle injuries [2,43]. Sudden increases in training volume or intensity are associated with a significantly higher risk of injury.

In a longitudinal study conducted on professional soccer players, Moreno-Perez et al. analyzed the relationship between the external match load profile and the occurrence of muscle injuries [56]. The results showed that injured players presented significantly lower values of total distance covered and high-speed running distance in the matches preceding the injury.

The Acute:Chronic Workload Ratio (ACWR) is used as a conceptual model to assess the relationship between recent training load and the athlete’s adaptive capacity [8,57]. Although exact thresholds vary across studies, the general consensus suggests that large fluctuations in workload increase the risk of muscle injuries. Although large fluctuations in workload have been associated with increased injury risk, the definition of these fluctuations is most commonly operationalized through the acute:chronic workload ratio. Previous literature has suggested that maintaining this ratio within a range of approximately 0.8 to 1.3 may represent a “sweet spot” associated with lower injury risk, whereas values exceeding this range may reflect rapid increases in load and heightened injury susceptibility [58]. However, these thresholds should be interpreted with caution, as the ACWR is influenced by the chosen calculation model, time windows, and sport-specific demands. Consequently, rather than representing a universal mathematical cutoff, ACWR should be considered a context-dependent indicator within a broader load management framework.

Neuromuscular fatigue negatively affects motor control, force production, and the capacity to absorb mechanical energy. The analyzed studies indicate that fatigue accumulated during competition and periods of high competition density constitutes a key determinant in the occurrence of muscle injuries [3,7,59].

## 5. Preventive Strategies and Biomechanics-Based Interventions

Modern strategies for the prevention of muscle injuries have evolved substantially in recent years, driven by advances in movement biomechanics, muscle physiology, and neuromuscular control. The analysis of literature published between 2010 and 2025 indicates that effective preventive interventions are those that directly target the biomechanical mechanisms responsible for injury occurrence, particularly eccentric loading at long muscle lengths, uneven force distribution, impaired neuromuscular control, and inadequate load management. The main preventive strategies discussed in the literature, together with their underlying biomechanical mechanisms and reported effects on muscle injury risk, are summarized in Table 1.

### 5.1. Eccentric Strength Training

The role of eccentric strength exercises in the prevention of muscle injuries is supported by a substantial number of experimental and interventional studies.

In a comprehensive biomechanical analysis published in *Strength and Conditioning Journal* in 2012, Cowell, Cronin, and Brughelli highlighted the central role of eccentric training in increasing the muscle’s capacity to absorb mechanical energy at long muscle lengths, thereby reducing local stress during deceleration and sprint phases [70]. The authors reported that exercises such as the Nordic hamstring and the Yo-Yo hamstring curl are associated with reductions in muscle injury incidence ranging between 30% and 60%, while the use of supramaximal loads (>100% of 1 RM) leads to significant increases in eccentric strength. The main conclusion was that strategically implemented eccentric training represents an effective tool for muscle injury prevention, provided that training volume and intensity are rigorously planned.

In a systematic review, Malliaropoulos et al. evaluated the effectiveness of eccentric exercises in hamstring injury prevention and reported reductions in incidence of up to 65%, along with significant decreases in recurrence rates [61]. The authors emphasized that the benefits are dependent on program adherence and the long-term integration of eccentric exercises.

In a meta-analysis conducted on professional and semi-professional soccer teams, Biz et al. demonstrated that injury prevention programs including eccentric exercises, such as the Nordic Hamstring Exercise, significantly reduce the incidence of hamstring muscle injuries [32]. Similarly, in a systematic review conducted by Ishøi et al. (2020), the authors concluded that there is strong evidence supporting the use of the Nordic Hamstring Exercise and the Copenhagen Adduction exercise as effective primary prevention strategies for lower limb muscle injuries in athletes [64].

At the elite sport level, Seagrave et al. conducted a prospective study published in the *Orthopaedic Journal of Sports Medicine* (2014), evaluating the effectiveness of the Nordic Hamstring exercise in preventing acute hamstring injuries among professional Major League Baseball players [18]. Monitoring 283 athletes over one competitive season demonstrated that none of the players who consistently followed the eccentric training program sustained a hamstring injury (0/65; 0%), compared with an incidence of 8.82% in the control group (3/34), a statistically significant difference. Furthermore, high compliance with the program was associated with a substantial reduction in injury severity, reflected by fewer total days lost, and the calculated number needed to treat (NNT = 11.3) underscored the practical effectiveness of the intervention.

Similarly, van der Horst et al. published in *Injury Prevention* the protocol of a multicenter randomized controlled trial designed to evaluate the efficacy of the Nordic Hamstring exercise in preventing hamstring injuries among amateur football players [23]. Conducted on a large sample (712 players from 38 teams), the study employed cluster randomization and a standardized protocol of 25 sessions over 13 weeks, with primary outcomes including injury incidence, recurrence, and severity. Based on existing evidence at the professional level, the authors estimated a potential reduction of approximately 70% in recurrent injuries, highlighting the translational relevance of validated biomechanical interventions to grassroots sport.

From a biomechanical perspective, Mendiguchia et al. used magnetic resonance imaging to analyze muscle loading distribution during the Nordic hamstring exercise, demonstrating predominant activation of the short head of the biceps femoris and the semitendinosus [71]. These findings suggest the need to complement eccentric exercises with hip-dominant movements to ensure comprehensive muscle injury prevention.

### 5.2. Neuromuscular and Integrative Training Programs

Neuromuscular and integrative training programs aim to optimize intermuscular coordination, dynamic stability, and postural control.

In a study conducted on professional football players, Owen et al. demonstrated that a multicomponent program including balance exercises, functional strength training, and core stability reduced the proportion of muscle injuries from 52% to 25%, corresponding to a relative reduction of 43% [72].

The authors emphasized that these exercises target the lumbopelvic–hip complex, including the abdominal muscles, spinal erectors, gluteal muscles, and hamstrings, thereby contributing to the optimization of lower limb biomechanics during sporting activities [8].

Similar results were reported in a systematic review conducted by Moncer et al. (2022), which analyzed the effectiveness of injury prevention programs for muscle injuries in soccer players. The authors showed that multimodal programs such as FIFA 11+ are particularly effective in reducing the incidence of severe muscle injuries, although their effect on minor injuries appears to be less pronounced [73].

In a study published in the *British Journal of Sports Medicine* in 2014, McCall et al. investigated current perceptions and practices regarding non-contact injury prevention in professional football through an international survey addressed to top-division clubs [17]. Analysis of responses from 44 clubs indicated that previous injuries, fatigue, and muscle imbalances were perceived as the main risk factors for injury, reflecting good alignment with existing epidemiological and biomechanical evidence. All participating clubs reported implementing prevention programs, with most utilizing combinations of individual and team-based interventions. Regarding applied strategies, eccentric exercises were considered the most effective by 79.5% of clubs, while 100% included core stability exercises in their programs, and 65.9% reported using the Nordic Hamstring exercise for hamstring injury prevention. However, the frequency of program implementation varied considerably depending on competition density, decreasing significantly during periods with two matches per week, suggesting potential underexposure to preventive stimuli precisely during high-risk periods.

At the youth level, McKay et al. examined psychosocial factors influencing adherence to the FIFA 11+ prevention program in a sample of female football teams [62]. Although the majority of coaches and players recognized inadequate warm-up as a risk factor for injury, only a small proportion believed that it could effectively prevent muscle or joint injuries, and these perceptions did not change significantly over the course of the season. Despite these knowledge gaps, adherence to the program was relatively high, particularly in groups receiving standardized and comprehensive implementation strategies. An important finding was the negative association between sporting experience and program adherence, both among coaches and players, suggesting that more experienced athletes may demonstrate greater resistance to standardized preventive interventions.

### 5.3. Core Muscle Strengthening and Lumbopelvic Stability

Lumbopelvic stability represents a fundamental biomechanical determinant in the prevention of musculoskeletal injuries, due to its role in maintaining segmental alignment and enabling efficient force transmission along the kinetic chain. In a review published in Sports Health in 2013, Huxel Bliven and Anderson analyzed epidemiological, biomechanical, and clinical evidence regarding core stability training and its relationship with injury risk [3]. The authors highlighted that deficits in trunk neuromuscular control, particularly delayed activation of local stabilizing muscles (transversus abdominis and multifidus), are associated with low back pain and an increased risk of lower extremity injuries. Prospective studies included in the review demonstrated that female athletes with poor trunk control had a 2.9-fold higher risk of anterior cruciate ligament injuries, and that multifactorial programs integrating core stability, balance, strengthening, and plyometric exercises could reduce injury incidence by up to 25% in female athletes and 85% in male athletes.

Complementarily, Fort-Vanmeerhaeghe and Romero conducted a review of neuromuscular risk factors in sports characterized by jumping, acceleration, and rapid changes of direction, emphasizing the importance of lumbopelvic stability and hip muscle activation as major modifiable factors in the prevention of non-contact injuries [1]. The authors reported that neuromuscular fatigue, altered muscle activation timing, increased dynamic valgus, bilateral asymmetries, and proprioceptive deficits are associated with an elevated risk of muscle and ligament injuries. Furthermore, fatiguing exercises were shown to reduce hamstring eccentric strength and increase mechanical loading at the knee joint, thereby amplifying injury risk.

### 5.4. Load Management and Fatigue

Load management represents an essential component of muscle injury prevention, particularly in sports characterized by high competition density and cumulative mechanical demands.

Hrysomallis reported that neuromuscular fatigue and previous injury history are the strongest predictors of muscle injury recurrence, and that preventive programs implemented under game-like conditions reduced hamstring injury incidence by 73% [2].

In a qualitative study, Saragiotto et al. showed that 83% of sports professionals perceived overload as the primary cause of muscle injuries; however, recovery and monitoring strategies were insufficiently utilized [74]. These findings highlight a gap between risk identification and the practical implementation of preventive interventions.

In a systematic review and meta-analysis of randomized controlled trials, Al Attar and Husain evaluated the effectiveness of injury prevention programs incorporating Core Muscle Strengthening Exercises (CMSEs) in reducing hamstring injury incidence among football players [8]. The analysis, which included data from 4728 players and over 379,000 h of exposure, demonstrated that integrating CMSEs into multimodal prevention programs leads to a significant reduction in muscle injury risk compared with conventional warm-up routines. The authors emphasized that strengthening the lumbopelvic–hip complex optimizes biomechanical control under high loading conditions, thereby reducing susceptibility to injury even during intense physical demands.

Complementarily, a recent systematic review examining the influence of high physical demands on the occurrence of muscle and ligament injuries in professional football players identified a direct relationship between competition density, high workload volume, and increased injury incidence, particularly during periods of congested match schedules [8]. The authors confirmed that cumulative overload and insufficient recovery are major determinants of non-contact injuries and identified the FIFA 11+ program as a viable preventive strategy capable of significantly reducing overall injury risk, including among young athletes.

### 5.5. Complementary and Emerging Interventions

In addition to the primary strategies based on eccentric training, neuromuscular control, and load management, certain complementary interventions may contribute to reducing muscular strain in specific contexts or may play an adjunctive role in recurrence prevention. In this regard, Chaudhari et al. investigated the effects of wearing directionally compressive shorts on muscle activation during rapid change-of-direction maneuvers [15]. The results demonstrated a significant reduction in electromyographic activation of the adductor longus throughout all phases of ground contact, without alterations in ground reaction force impulse or overall biomechanical performance. These findings suggest that compressive interventions may reduce localized muscular loading during complex dynamic tasks, with potential utility in preventing adductor recurrences or during progressive return-to-play phases; however, they do not replace active training strategies.

In an experimental study conducted on soccer players, Kamandulis et al. (2022) evaluated the effectiveness of a high-velocity training program for the hamstring muscles performed using elastic resistance bands [50]. Although the results indicated an approximately 30% reduction in injury risk, the intervention did not demonstrate statistically significant effectiveness, suggesting the need to integrate this method into more comprehensive injury prevention programs.

With respect to stretching, Stojanovic and Ostojic conducted a critical review of the available literature regarding its role in muscle injury prevention in football [13]. The authors reported that static stretching protocols can temporarily reduce muscle–tendon unit stiffness, with decreases ranging from 8% to 47%; however, these effects are transient and dissipate within approximately one hour. Although epidemiological studies suggest that reduced flexibility is associated with an increased risk of muscle injury, the evidence does not support isolated stretching as a robust preventive strategy. Instead, the benefits appear to depend on the type, duration, and frequency of stretching exercises and are more consistent when stretching is integrated into multifactorial programs alongside eccentric strength training and neuromuscular conditioning.

### 5.6. Technological and Predictive Approaches

In an experimental study based on IoT technologies, the authors demonstrated that intelligent data collection systems can identify movement patterns associated with an increased risk of injury and can optimize prevention and rehabilitation programs [43].

Similarly, Yang et al. (2023) developed a stretchable surface electromyography patch capable of monitoring muscle activity, fatigue, and tendon displacement in real time [75]. The authors suggest that this technology may contribute to reducing the risk of muscle and tendon injuries through continuous monitoring of biomechanical loads.

In parallel, modern data analysis methods have begun to be used for the prediction of muscle injuries. In a recent study based on machine learning algorithms, the authors demonstrated that the XGBoost model can predict injury risk in professional soccer players with an accuracy of approximately 78%, identifying maximum hamstring strength and stiffness as important biomechanical predictors [53].

In a recent experimental study focused on wearable haptic technologies, researchers developed a forearm device based on artificial muscles capable of delivering controlled tactile feedback with high precision and responsiveness. The system demonstrated reliable performance in reproducing force patterns associated with muscle activity and external interaction. The authors suggest that such technologies may enhance neuromuscular control and motor learning through real-time feedback, with potential implications for injury prevention and rehabilitation. These findings highlight the growing role of bioengineering solutions in the development of individualized and adaptive training strategies [76].

## 6. Discussion

Muscle injuries remain one of the most common causes of time loss in both recreational and professional sports, highlighting the importance of effective preventive strategies. The literature reviewed confirms that these injuries result from a multifactorial interaction between sport-specific biomechanical demands, neuromuscular control capacity, and the adaptive limits of the muscle–tendon unit. Variations across sports disciplines, competition levels, and exposure contexts suggest that muscle injuries should be understood as the consequence of complex interactions between mechanical loading, movement control, and tissue adaptation.

Biomechanical analyses consistently identify the muscle–tendon unit as particularly vulnerable during eccentric contractions performed at long muscle lengths, conditions commonly encountered during sprinting, deceleration, and rapid changes of direction. These situations generate high mechanical stress on muscle fibers and connective tissues, explaining the predominance of hamstring injuries in sports involving explosive movements. However, much of this evidence is derived from controlled experimental settings, which may limit its direct applicability to real-world sporting conditions, where additional factors such as fatigue and individual technical variability play a significant role. In addition to maximal force production, muscle architecture—particularly fascicle length and muscle–tendon stiffness—plays an important role in determining the capacity of muscle tissue to tolerate mechanical strain.

Strength imbalances also represent a relevant determinant of injury risk. The hamstrings-to-quadriceps (H:Q) strength ratio, especially the eccentric component of hamstring strength, contributes to functional stability of the knee during dynamic movements. Reduced H:Q ratios and bilateral asymmetries may alter load distribution during high-intensity actions, increasing mechanical stress on vulnerable muscles. These imbalances become more critical under conditions of neuromuscular fatigue, when both force production and coordination decline. Consequently, isolated strength assessments may not fully reflect the functional complexity of injury risk.

Despite the strong body of evidence supporting the efficacy of the Nordic Hamstring Exercise in reducing the incidence of muscle injuries, the transfer of these benefits into real-world practice largely depends on athlete adherence. The literature suggests that adherence to general preventive exercise programs may reach approximately 85%, whereas adherence to targeted interventions such as the Nordic Hamstring Exercise has been reported at around 69% among amateur football players, indicating a more challenging implementation of high-intensity eccentric protocols. Furthermore, observational data have shown that, despite its demonstrated effectiveness, most high-level football teams do not systematically adopt this type of intervention, highlighting a significant gap between experimentally demonstrated efficacy and real-world applicability. Therefore, the preventive impact of eccentric training should be interpreted not only in terms of injury risk reduction, but also in relation to implementation feasibility, protocol tolerability, and the sustainability of athlete participation.

Neuromuscular control and lumbopelvic stability play a central role in injury prevention. Deficits in proximal stability, such as delayed activation of trunk stabilizers or insufficient hip muscle control, may lead to compensatory strategies at the level of the lower limbs. These compensations can increase mechanical loads on muscles such as hamstrings or adductors during dynamic tasks. From a biomechanical perspective, the trunk and pelvis act as key components of the kinetic chain responsible for transmitting forces across body segments.

Load management and neuromuscular fatigue represent additional factors influencing injury occurrence. High training volumes, dense competition schedules, and inadequate recovery may exceed the adaptive capacity of the musculoskeletal system. Sudden increases in workload can contribute to the accumulation of microtrauma and deterioration of neuromuscular control. However, the absence of clearly defined quantitative thresholds limits the practical applicability of these concepts in both clinical and sports settings. Therefore, preventive strategies must be integrated into training periodization, as their effectiveness depends not only on the type of exercises performed but also on their timing relative to competition cycles and fatigue levels.

Among the preventive interventions analyzed, eccentric strength training—particularly through the Nordic Hamstring Exercise—emerges as one of the most effective strategies for reducing muscle injury risk. Its protective effects appear to involve structural adaptations, including increases in fascicle length and improved capacity of the muscle–tendon unit to absorb mechanical energy during eccentric loading. Nevertheless, its effectiveness in real-world settings may be influenced by athlete compliance, which has been reported as variable, particularly in high-intensity eccentric protocols. Moreover, the optimal intensity and dosage of such interventions may vary considerably between athletes, depending on individual factors such as training level, previous injury history, and neuromuscular capacity.

Integrated neuromuscular training programs, such as FIFA 11+, further highlight the importance of multifactorial prevention strategies. By combining balance exercises, functional strength training, and dynamic stability drills, these programs improve intermuscular coordination and movement control during sport-specific tasks.

Core muscle strengthening represents another component of integrated prevention strategies. Improvements in lumbopelvic stability may indirectly reduce the risk of lower-limb injuries by optimizing force transfer across body segments and stabilizing the trunk during dynamic movements.

Complementary interventions such as compression garments or stretching may provide additional biomechanical benefits in specific contexts, but current evidence does not support their use as standalone preventive strategies. Their effects appear limited unless they are integrated into broader multimodal training programs addressing the primary biomechanical determinants of injury risk.

Another important issue highlighted by the literature is the gap between scientific evidence and the practical implementation of preventive programs in sports environments. Even interventions supported by strong evidence are not consistently applied across teams or competition levels, often due to time constraints, competition schedules, or athlete perceptions. The interpretation and comparison of findings across studies investigating muscle injury prevention are often limited by the lack of standardized definitions and heterogeneous data collection methodologies. In this context, the International Olympic Committee has emphasized the importance of adopting consistent frameworks for injury and illness surveillance, including uniform definitions, classification systems, and reporting procedures, in order to enhance the comparability and quality of epidemiological data. The IOC consensus statement highlights that variations in injury definitions and differences in data collection approaches can lead to substantial discrepancies in reported incidence and risk patterns across studies. Therefore, the implementation of standardized reporting guidelines, such as the STROBE extension for sport injury and illness surveillance, represents a critical step toward reducing methodological heterogeneity and improving the reliability and clinical applicability of research findings in the field of sports injury prevention.

Recent technological developments may contribute to improving injury prevention strategies. Wearable sensors and electromyography systems enable continuous monitoring of biomechanical variables during training and competition. Combined with machine learning techniques, these technologies may help identify patterns associated with injury risk and support the development of individualized prevention strategies.

Although the reviewed literature highlights numerous biomechanical and neuromuscular factors involved in the occurrence of muscle injuries, the translation of these findings into applicable strategies remains limited by the fragmented nature of existing studies. In this context, an integrated prevention approach should include the initial assessment of sport-specific risk factors, the identification of functional imbalances and neuromuscular deficits, as well as the implementation of interventions targeting eccentric training, neuromuscular control, lumbopelvic stability, and load management. However, these components are often examined separately in the current literature and are not unified into standardized protocols, which limits their direct applicability in practice. Therefore, the findings of the present review may serve as a starting point for the development of integrated prevention strategies tailored to specific sports and performance levels.

Overall, the evidence synthesized in this review indicates that muscle injury prevention should be approached as a multifactorial process grounded in biomechanical and neuromuscular principles. However, methodological heterogeneity and the lack of standardized frameworks limit the robustness of current conclusions, highlighting the need for future research that is more standardized and clinically oriented. Effective prevention requires the integration of eccentric strength training, neuromuscular coordination exercises, lumbopelvic stability training, and appropriate workload management in order to reduce injury risk and support long-term athletic performance.

## 7. Conclusions

Muscle injuries represent a major challenge in both recreational and professional sports due to their high incidence and recurrence rates, with significant consequences for athletic performance and career longevity. The evidence synthesized in this review indicates that the development of these injuries results from a complex interaction between sport-specific biomechanical demands, neuromuscular control capacity, and the adaptive properties of the muscle–tendon unit.

Key biomechanical factors associated with muscle injury risk include eccentric contractions performed at long muscle lengths, strength imbalances between agonist and antagonist muscle groups, deficits in lumbopelvic stability, and the accumulation of neuromuscular fatigue. Effective preventive strategies should therefore move beyond isolated interventions and target the fundamental biomechanical mechanisms underlying injury occurrence.

Eccentric strength training, integrated neuromuscular programs, and core muscle strengthening have demonstrated consistent effectiveness in reducing both the incidence and severity of muscle injuries, highlighting the importance of biomechanically informed prevention strategies. In addition, appropriate workload management and the integration of preventive interventions within training periodization remain essential for optimizing their effectiveness.

Future research should focus on the development of predictive models and individualized prevention strategies that integrate biomechanical assessment, technological monitoring systems, and data-driven approaches. Such advances may contribute to more precise injury risk identification and to the optimization of preventive programs in modern sport.

## Figures and Tables

**Table 1:** Summary of biomechanical mechanisms and preventive interventions for sports-related muscle injuries.

Sport/Context	Predominantly Affected Muscle Group	Dominant Biomechanical Mechanism	Preventive Interventions Investigated	Reported Effects
Professional & Amateur Soccer [1,5,17,18,23,46,58,60,61,62]	Hamstrings, adductors	Eccentric contractions at long muscle lengths; cumulative fatigue; H:Q imbalances	Nordic Hamstring Exercise, FIFA 11+, multifactorial programs	40–70% reduction in incidence; decreased recurrence rates
Australian Football [2,32,55,63]	Hamstrings, thigh musculature	Overuse, altered muscle stiffness, impaired neuromuscular control	Eccentric training, core stability training, game-specific conditioning	Hamstring injury reduction up to 73%
Track & Field (Sprint Events) [39,55,64,65]	Hamstrings	Maximal elongation combined with eccentric activation during terminal swing phase	Nordic Hamstring Exercise, hip-dominant eccentric exercises	~65% reduction in incidence; decreased recurrence
Baseball (Pitching & Sprinting) [15,17,66]	Hamstrings, elbow flexor–pronator complex	Repetitive eccentric overload; dynamic instability	Nordic Hamstring Exercise; forearm flexor–pronator strengthening	0% vs. 8.82% hamstring injury rate; reduced UCL stress
Basketball Volleyball [4,10,20,35]	Quadriceps, adductors, knee stabilizers	Repetitive landing; dynamic valgus; bilateral strength asymmetries	Integrative Neuromuscular Training (INT), Core Muscle Strengthening Exercises (CMSE), selected closed kinetic chain exercises	Reduced harmful joint loading; improved neuromuscular activation balance
Swimming [67]	Shoulder, scapular stabilizers	Repetitive overuse; scapulohumeral imbalance	Scapular strengthening + stretching protocols	Prevention of strength decline; limited kinematic modification
Cutting/Change-of-Direction Sports [15]	Adductors	Increased mechanical demand during run-to-cut maneuvers	Directional compression garments	Reduced EMG activation without performance impairment
Recreational Winter Sports [56,68]	Lower extremity (general)	Extrinsic factors; insufficient conditioning	Equipment modification; education-based prevention	Major gap in exercise-based preventive strategies
Multisport (General) [3,29,69]	Trunk, hip, hamstrings	Impaired neuromuscular control; reduced lumbopelvic stability	Core Muscle Strengthening (CMSE), multifactorial prevention programs	25–85% reduction in injury incidence

## Data Availability

The original contributions presented in the study are included in the article, further inquiries can be directed to the corresponding author.
